# The Interplay of Achievement Motive-Goal Incongruence and State and Trait Self-Control: A Pilot Study Considering Cortical Correlates of Self-Control

**DOI:** 10.3389/fnbeh.2019.00235

**Published:** 2019-10-04

**Authors:** Julia Schüler, Jonas Hofstetter, Wanja Wolff

**Affiliations:** ^1^Department of Sport Science, University of Konstanz, Constance, Germany; ^2^Institute of Educational Sciences, University of Bern, Bern, Switzerland

**Keywords:** achievement motive, motive-goal incongruence, self-control, motor performance, fNIRS

## Abstract

**Objective**: This study utilized different theoretical perspectives to better understand motor performance. We refered to concepts of achievement motive-goal incongruence and assessed cortical correlates of self-control. We assumed that more self-control is required when people act in conformance with an incongruent goal which, in turn, results in impaired performance. We considered the activation of a brain area associated with self-control (dorsolateral prefrontal cortex, dLPFC) as a consequence of motive-goal incongruence. Furthermore, we analyzed whether trait self-control buffers the negative effects of achievement motive—goal incongruence.

**Method**: Twenty-eight participants (17 women, mean age: 24 years), whose implicit achievement motives were assessed at the beginning of the study, performed a handgrip task in an achievement goal condition and in three incongruent conditions, while their dLPFC oxygenation was monitored continuously (using functional near-infrared spectroscopy, fNIRS).

**Results**: None of the two-way interactions (motive × goal condition) reached significance. A significant three-way interaction (motive × trait self-control × goal condition) showed that trait self-control buffered the detrimental effects of incongruence on motor performance. The nature of the three-way interaction predicting dLPFC oxygenation was unexpected.

**Conclusions**: Although our results have to be treated with caution due to a small sample size, we see them as an encouraging starting point for further research on the interplay between motive-goal incongruence and trait and cortical correlates of state self-control that we assume to be important to understand performance in strenuous tasks.

## Introduction

Previous research has convincingly demonstrated that people’s well-being and motivation are impaired when they pursue goals in their daily lives that do not fit their personality (Sheldon and Elliot, 1999). One aspect of personality is implicit motives which are defined as stable preferences for certain kinds of incentives (Atkinson, 1957; McClelland et al., 1989). For example, people with strong implicit achievement motives chronically prefer challenging performance tasks because they anticipate feeling proud when having mastered these tasks and when having improved their skills. However, satisfying ones’ motives requires setting the right goals. If an achievement motivated person repeadedly pursues goals that are unrelated to achievement (e.g., relationship goals) their implicit achievement motive cannot be satisfied. This non-fit is called motive-goal incongruence and it is detrimental to well-being (Brunstein et al., 1998, 1999; Michalak et al., 2006; Schüler and Wegner, 2015; Schüler et al., 2017). Researchers agree that pursuing motive-incongruent goals requires effort and mental resources (Brunstein, 2010a) and therefore hinges on self-control. However, studies that examine exactly *how* motive-goal incongruence is associated with self-control are rare (for an exception, see below; e.g., Kehr, 2004b). Here, we provide a first transdisciplinary study to address this research gap.

In the following paragraphs we outline the concepts of motive-incongruence and self-control and suggest two mechanisms on how they are linked: first, we assume that pursuing motive-incongruent goals requires more state self-control than pursuing goals that are congruent with one’s motives. Second, trait self-control is assumed to influence how motive-goal incongruence exerts its effects on state self-control and on motor performance.

Research on motive-goal incongruence has primarily assessed detrimental effects on well-being and its’ potential effects on performance have been neglected so far. Here, we assess the effect of motive-goal incongruence on motor performance. One aspect of motor performance is performance in strength endurance tasks such as the hand grip tasks that assess the isometric strength of hand and forearm. Handgrip strength is a useful measure because it is frequently used in lab research to assess self-controlled behavior and it serves as a diagnostic indicator in more applied settings that range from clinical rehabilitation to sports performance. Due to our interest in motor performance, we focused on the achievement motive (rather than on the affiliation or power motive, see below), which is defined as the desire to manage challenging tasks (Brunstein and Heckhausen, 2008).

Before we get into details, we would like to draw the readers’attention to the fact that the present study is a pilot study that we used to link complex theoretical frameworks from motive research (motive incongruence and their interplay with self-control) with neuroscientific approaches: we used oxygenation of the dorsolateral prefrontal cortex (dLPFC) as an operationalization of self-control. We invite the readers to think critically about our application of neuroscientific measures (functional near-infrared spectroscopy, fNIRS) to complex, mainly theoretically-based psychological considerations.

### Motive-Goal Incongruence and Its Consequences

The concept of motive incongruence is based on the assumption that two motivational systems—an implicit and an explicit motivational system—exist that operate independently from each other and therefore can be more or less congruent (McClelland et al., 1989).

*Implicit motives* represent the “capacity to experience the attainment of a certain type of incentive as rewarding […]” (Schultheiss and Hale, 2007, p. 13). Compared to people with a low implicit achievement motive, people with a high achievement motive experience more pride and better well-being as a consequence of having mastered a challenging task. As a consequence, the latter are “[…] oriented towards cues related to the incentive and energizes, and selects behavior aimed at incentive attainment” (Schultheiss and Hale, 2007, p. 13). In brief, motives are triggered by incentives that are relevant for motive satisfaction which in turn energizes and directs their behavior. On the example of the achievement domain, the achievement motive is triggered by tasks of moderate difficulty, and the announcement of feedback about one’s performance, which in turn creates the urge to behave in a certain kind of way (e.g., giving ones’ best to master a challenge). Motive arousal happens automatically (McClelland, 1985a) and the operating principles of implicit motives are thought to evade conscious awareness, thereby making them inaccessible to introspection. Consequently, implicit motives are not assessed with traditional self-report measures but with indirect measures like picture story exercises (Schultheiss and Pang, 2007; for other measures of implicit motives, see Operant Motive Test, OMT; Kuhl and Scheffer, 1999).

Implicit motive researchers have mainly focused on three motives that capture different content domains and that are assumed to explain the broad range of human cognition, emotion and behavior very well (McClelland, 1985b; Schultheiss and Brunstein, 2010). In addition to the *achievement motive*, which is defined as a recurrent concern with surpassing standards of excellence (McClelland et al., 1953) and which is incited by challenging tasks that promise successful mastery and the experience of competence (Schultheiss, 2008, p. 608), two social motives have been examined: the *affiliation motive* (recurrent concern with building up and maintaining stable and friendly interpersonal relations, French and Chadwick, 1956; Sokolowski, 2010) and the *power motive* (desire to influence and control other people in order to feel strong and superior to others, Winter, 1973; McClelland, 1985b; Fodor, 2010).

*Goals* are part of an *explicit, consciously-represented motivational system* (McClelland et al., 1989). They represent conscious and rational choices of what one deems important to strive for in life. In contrast to implicit motives, which tend to be aroused by affective incentives promising rewarding emotions, goals are elicited by rational incentives including social expectations, demands, and external rewards. A person might pursue an achievement goal (My goal is to succeed in the exam; I want to win the gold medal) in order to fulfill the expectations of key persons (significant others, parents, coaches, peers), because of the rewards associated with goal attainment (academic career, prize money) and because of the underlying self-concept of being an achievement-oriented person (I am a person for whom achieving high standards of excellence is important). The self-concept is based on conscious reflections (What person am I?), whereas the implicit motive is based on affects (anticipating satisfaction while pursuing an achievement goal feeling proud after goal attainment).

The assumption that the implicit and explicit motivational systems operate independently from each other (McClelland et al., 1989), is supported by meta analyses showing statistical independence of both motivational systems (Spangler, 1992; Köllner and Schultheiss, 2014). This statistical independence indicates that the implicit and explicit motivational system can be more or less overlapping or, in other terms, can be more or less *congruent* or *incongruent*. Motive-goal incongruence has severe costs, particularly for ones’ emotional well-being. Several studies have shown that motive-goal congruence is positively and motive-goal incongruence is negatively associated with emotional well-being (Brunstein et al., 1995, 1998; Hofer and Chasiotis, 2003; Baumann et al., 2005; Schüler et al., 2009; Hofer et al., 2010; Job et al., 2010; Langan-Fox and Canty, 2010; Pueschel et al., 2011; Hofer and Busch, 2017).

### Linking Motive-Goal Incongruence and Self-Control

Theoretical considerations why motive-incongruence unfolds its’ negative effects largely conceptualize motive-goal incongruence as a “psychological conflict” (Kehr, 2004a,b) and a “hidden stressor” (Baumann et al., 2005) that arouses incompatible behavioral tendencies derived by the implicit motive and the explicit goal (McClelland et al., 1989). One example is a student who feels a strong urge to master challenging academic tasks (implicit achievement motive), but who committed himself to build up a social network at his new place of studies (explicit affiliation goal). Should he stay at home and continue reading further attractive scientific articles or should he break himself away from studying and go to a party in order to meet people? (Depending on his or her own motive scores, the reader might be better able to imagine the opposite conflict). Resolving this conflict between incompatible behavioral tendencies elicited by implicit motives and explicit goals conceivably relies on self-control, which has been defined as “the ability to override or change one’s inner responses, as well as to interrupt undesired behavioral tendencies (such as impulses) and refrain from acting on them” (Hofmann et al., 2014, p. 265). More specifically, motive-goal incongruence is very similar to what researchers have coined a self-control dilemma: “situations in which competing behavioral tendencies create a conflict that needs to be resolved” (de Ridder et al., 2018, p.39). In brief, we consider motive-goal incongruence as a conflict that requires self-control.

Although many researchers agree that self-control is needed to counteract the conflict between implicit and explicit motivational systems (Kuhl, 2001; Brunstein, 2010a), self-control has rarely been measured. One exception is Kehr’s (2004a) longitudinal field study with managers. He assumed that the non-fit between the implicit and explicit motivational system leads to a psychological conflict and that the resolution of this conflict requires self-control. In this study, the managers’ implicit motives were assessed using a semiprojective diagnostic tool in order to capture the unconscious nature of implicit motives (Multi-Motive Grid, Sokolowski et al., 2000). The explicit motivational concept was assessed with a questionnaire (subscales of the Personality Research Form, Stumpf et al., 1985). The extent of self-control that the managers had to mobilize in their daily lives was assessed by a self-control questionnaire (Volitional Component Inventory, Kuhl and Fuhrmann, 1998; item example: “If I want to, I am able to deliberately concentrate on whatever is important at the moment”). As expected, the incongruence between the implicit and explicit motivational system impaired participants’ well-being across a time period of 5 months. Kehr’s further analyses showed that self-control mediated the relationship between motive incongruence and well-being. These results confirmed not only that acting incongruent to ones’ implicit motives reduces well-being, but also supported the assumed underlying mechanism that incongruence impairs well-being because it imposes high self-control demands. A limitation of the study is that the self-control measure solely relied on self-reports, a fact that the author himself discussed critically (p. 325). Kehr (2004a, p. 325) stated that “It is difficult to assess internal, action-related conflicts and volitional conflict resolution strategies without self-report.” We agree that self-control would better be assessed using a more indirect measure that is more suitable to address implicit processes. Therefore, in the present study we focus on a cortical correlate of self-control.

#### Cortical Correlates of Self-Control

Neuroscientific research that examined which brain regions are mainly active during self-control exertion has convincingly shown that the PFC is involved in detection and control of conflicts (Carter and van Veen, 2007; Cohen and Lieberman, 2010). Results suggest that the dorsal anterior cingulate cortex (dACC) seems to detect conflicts, whereas the dLPFC is more involved in processes that are needed to manage the conflict, for example by impulse control (Botvinick et al., 2001, 2004; Shenhav et al., 2017). Research from cognitive neuroscience has provided empirical support for the dLPFC’s role in impulse control. The Stroop task, for example, constitutes situations in which competing behavioral tendencies (e.g., naming the color of the word vs. reading the word) create a conflict (León-Carriona et al., 2008). Also studies in the domain of sports that for example examined self-control requirements of a sprint start (Wolff et al., 2019) found activation increases in the dLPFC when impulse control demands were high. To come back to the concept of motive-goal incongruence, we referred to the empirical support for dLPFC-activation as an indicator of impulse control to our research and assumed that the dLPFC is more activated when people need to override automatically derived behavioral tendencies of their implicit motive and instead have to act in accordance with an incongruent goal. In brief, our first approach to link motive incongruence with self-control demands is assuming the latter to be a consequence of the former.

#### Trait Self-Control as a Moderator

Self-control has been conceptualized as a state and a trait (Tangney et al., 2004) and our second approach to link motive incongruence and self-control focuses on *trait self-control*: “the stable ability to handle self-control dilemmas in such a way that the desired goal is prioritized” (de Ridder et al., 2018, p. 49). We propose that trait self-control is a moderator which influences the effects of motive incongruence on performance and state self-control. We focused on performance as an outcome measure of motive incongruence because studies testing the effects of motive-goal incongruence on performance are relatively rare (for exceptions, see Schultheiss and Brunstein, 1999; Schüler et al., 2017), especially when compared to numerous studies addressing well-being (see above). One reason for the less clear empirical link between incongruene and performance might be that other variables also have a strong impact on performance (e.g., skill level) and therefore might overshadow the effects of motive-incongruence. Trait self-control is an example of a variable that has a strong impact on both cognitive performance (Duckworth and Seligman, 2005) and motor performance (Bray et al., 2015; Taylor et al., 2018). In contrast to state self-control that is susceptible to situational influences and varies across situation and time (Tangney et al., 2004) (e.g., as a response to a motive incongruent goal), trait self-control is conceptualized as a relatively stable characteristic of a person (de Ridder et al., 2012).

Trait self-control is linked with a variety of positive outcomes (e.g., de Ridder et al., 2012), among them performance in academia (Tangney et al., 2004; Duckworth and Seligman, 2005), at the work place (de Ridder et al., 2012) and in sports (Englert, 2017). Furthermore, people with high trait self-control are better able to deal with situational self-control requirements (Gillebaart et al., 2016; de Ridder et al., 2018). We assume that participants with high trait self-control might also better be able to cope with motive-goal incongruence in terms of less impairment of their motor performance. Additionally, for exploratory reasons, we examined the role of trait self-control when predicting dLPFC-oxygenation in motive-incongruent situations.

### Present Research and Hypotheses

We used a within-subject study design in order to induce goals that were either congruent or incongruent with an implicit achievement motive. Therefore, participants received written instructions to perform an identical motor task but we experimentally varied the content of this instructions. Participants were successively assigned to an achievement motive congruent goal condition (achievement goal; aim of task performance: exceed one’s standard of excellence) and to different types of goal conditions that are incongruent with the achievement motive. The incongruent *power goal* (aim of task performance: demonstrating one’s superiority) and *affiliation goal* (aim of task performance: committing oneself to a peer group) are associated with incentives that are relevant for other motives, and therefore create a conflict with the implicit achievement motive. Resolving this conflict supposedly requires self-control. In contrast, the *motive-neutral goal* (aim of task performance: calibration of a technical device) is not associated with any motive-relevant incentive. Incongruence in this case means a lack of achievement incentives, which is assumed to require extra effort (and therefore more self-control) for people with a strong achievement motive (McClelland et al., 1953; McClelland, 1987). We assumed that for them motive-incongruent goals require more self-control and that these higher self-control requirements should be measurable in stronger dLPFC oxygenation. Summing up, we hypothesized that participants with a strong achievement motive show lower dLPFC oxygenation in the achievement goal condition than in the motive incongruent goal conditions (affiliation, power and motive-NEUTRAL goals). We used fNIRS as a non-invasive imaging method to visualize the expected activity changes in the dLPFC (for details, see below). We furthermore tested whether and how trait self-control functions as a moderator of this effect (three-way interaction) for exploratory reasons. We did not formulate a directed hypothesis, because from a theoretical point of view it is not clear how trait self-control contributes to successful goal striving in this case. Trait self-control is known to make state self-control available when it is needed (Gillebaart et al., 2016; de Ridder et al., 2018). In our study, however, we refer to the theoretical background of motive incongruence research and evaluated the self-control requirements of congruent and incongruent goals and therewith, strictly speaking, characteristics of the type of goal rather than the availability of self-control.

Regarding our second approach to examine how motive-goal incongruence and trait self-control interact to predict performance, we chose motor performance (isometric hand strength) as the performance outcome measure, because it can be carried out easily in the laboratory (in which the fNIRS measure took place), and because it can easily be adapted to various goal conditions. Referring to previous research on motive-goal incongruence in sport (Schüler et al., 2017), we hypothesized that participants with a strong achievement motive perform better in a motor performance task (hand-grip task) when being assigned to the motive-congruent achievement goal condition than when being assigned to motive-incongruent goal conditions (affiliation, power, and motive-NEUTRAL goals). In sum, we expected a two-way interaction effect (motive × goal condition) on motor performance.

Furthermore, we hypothesized that trait self-control can outweigh the negative effects of motive-goal incongruence on motor performance and tested for a three-way interaction effect on motor performance.

## Materials and Methods

### Participants and Procedure

**Figure 1:**
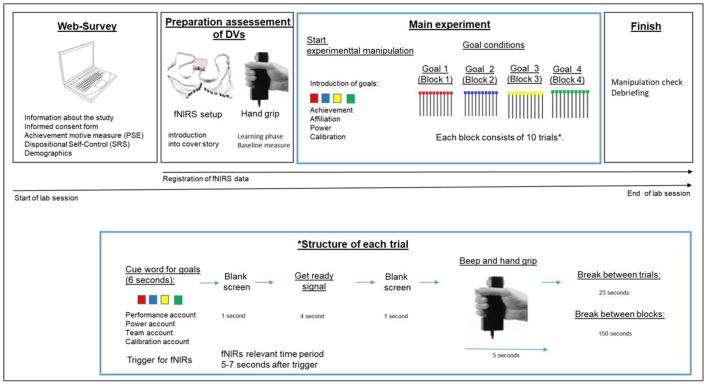
Structure of data collection in lab session (upper part) and zoom into the time flow of one trial in the main experiment.

Twenty-eight students (11 men, 17 women) with a mean age of 24 years (*SD* = 3.15) from a German University participated in a study that was advertised as a study about creativity and motor performance. A university-internal platform was used for the recruitment of participants who received 20 EUR for a total of 2 h of study participation. We based our study on a randomized, experimental within-subject design in order to enhance statistical power. The study procedure is depicted in Figure 1 and will be outlined in the following. We experimentally-induced achievement, affiliation and power goals as well as a motive-neutral goal in separate blocks (block order was randomized for participants) and assessed hand grip strength as the dependent variable 10 times in each block. The study design and the debriefing form met the standards of Ethics Committee of the authors’ university and were in line with the Declarations of Helsinki (World Medical Association, 2013).

At the beginning of the experiment, participants filled in a web-survey containing an information sheet informing participants that the study is about cortical activation during a strenuous motor task. The informed consent form was provided as a paper version. Then, back to the web-survey, participants filled in the implicit achievement motive measure (Picture Story Exercise, PSE, Morgan and Murray, 1935; Schultheiss and Pang, 2007), and additionally the dispositional self-regulation scale (SRS, Schwarzer et al., 1999). The survey ended with asking participants to enter their age and gender. Then, the experimenter assessed participants’ head circumference, chose a fNIRS cap and a cover cap and prepared the dLPFC optode montage. After a signal quality check (and a readjustment of optodes if necessary), participants were asked to take a comfortable sitting position and follow the instruction on a computer screen that would lead them through the following experiment.

During the fNIRS setup, participants read a cover story that introduced the experimental manipulation, which followed later. Participants were told that, because fNIRS is a time-consuming and expensive technology, not one, but three master students with related research questions—all about creativity and motor performance—would collect data for their master theses. These alleged three master theses functioned as an explanation to improve plausibility of the three experimental conditions that followed later. Then the experimenter explained the motor task. Participants learned that they have to press the hand-grip as hard as possible during the period of a beep-signal. After having ensured that participants understood how to use the handgrip, the experimenter started the baseline measure for hand-grip strength^1^.

In order to reduce the experimenter’s influence in the subsequent main experiment, PsychoPy (Peirce, 2007) was used to instruct participants and guide them through the experiment. This script included more study information about the already introduced master theses that in fact represent the achievement, affiliation, power and neutral goal conditions (for details, see experimental manipulation below) and continued with the four related blocks (experimental conditions: achievement goal, affiliation goal, power goal, motive-neutral goal) with 10 trials for each goal condition. Each trial started with a cue-word, which lasted 6 s (reasons for chosen time-intervals, see below) and reminded participants of the specific goal. The cue-word was followed by 1 s of blank screen and, subsequently, by a brief instruction on the computer screen (lasting 4 s) that announced the beep signal and prompted participants to ready themselves to press the hand grip dynamometer. Another second of blank screen was followed by the beep signal (lasting 5 s) and participants had to press the hand-grip. After a break of 23 s, the next trial started. The breaks between blocks lasted 2 min and 30 s. At the end of the main experiment, participants filled in the manipulation check form and were fully debriefed by the experimenter.

### Experimental Manipulation

The cover story (i.e., three different master theses) was introduced during the fNIRS setup. Participants were told that data for three different master theses based on research questions about creativity, cortical activity and motor performance were collected. One thesis supposedly was about individual performance (achievement framing to introduce the achievement goal, see below), another about the ranking of performance and comparison between students (power framing) and another supposedly was about team performance (for similar motive-relevant framing in motor tasks, see for example Sorrentino and Sheppard, 1978; Schüler et al., 2017). This information prepared the experimental manipulation that started at the beginning of the main experiment. In the main experiment, participants were informed that they have to perform a task in different conditions (the “blocks,” see Figure 1) that were related to the master theses introduced before.

In the *achievement goal condition*, they will receive a summary sheet about their individual motor performance level and their performance development at the end of the experiment. This announcement of feedback is expected to serve as a strong achievement incentive (see for example McClelland, 1985a). The paragraph that frames achievement ends with a specific achievement goal that refers to an individual reference standard of performance: “Do your best to optimize your individual performance and fill your *performance account*.” The term *account* was chosen because participants were told that by pressing the hand grip they can collect points for the respective account (i.e., separate “accounts” per alleged master theses). The term *indiviudal* p*erformance account* was used as a cue word prior to each trial in the achievement goal block of the main experiment in order to remind participants of the achievement goal.

In the *power goal condition*, participants learned that they have to collect points that determine their position on a ranking list of participating students. The ranking list was announced to be published so that they can see the students they had outperformed and vice versa. This power framing ended with the assigned goal “Do your best to demonstrate your performance superiority and fill your *power account*.”

In the *affiliation goal condition*, participants were told that they were expected to collect points for a team that consists of students who are similar to themselves (with regard to creativity scores, performance, demographic characteristics). A fact sheet about the similarities between members of a group was announced to be published for the ingroup members at the end of data collection. The affiliation goal was “Do your best for your team and fill your *team account*.” In the motive-neutral condition, participants were told that the hand-grip device had to be calibrated in order to control for unwanted drifts caused by the device and that this also required to press the hand-grip as hard as possible. The cue word for this condition was *calibration* and the specific goal was “Do you best for the calibration.”

### Measures

We assessed participants’ *implicit achievement motive* at the beginning of the laboratory session using the Picture Story Exercise (PSE, Schultheiss and Pang, 2007), which is a reliable, valid and commonly used test in implicit motive research (Schultheiss et al., 2008; Schüler et al., 2015). Participants write imaginative stories to six pictures that are then scored by the experimenter using Winter’s scoring manual for motive imagery in running text (Winter, 1994). In our study, the stories were coded by a coder with extensive coding experience (according to criteria suggested by Schultheiss and Pang, 2007) for the achievement, affiliation and power motive. Winter’s scoring manual contains precise rules for scoring participants’ written stories and specifies scoring categories for the motives. The achievement motive, for example is scored for any indication of a standard of excellence that is expressed in five forms including: (1) adjectives that positively evaluate performance; (2) goals or performances that are described in ways that suggest positive evaluation; (3) mention of winning or competing with others; (4) failure, doing badly, or other lack of excellence; and (5) unique accomplishment (Winter, 1994, p. 10–11). The sum of all indications across all six pictures results in an overall achievement motive score (The same applies to the categories for the affiliation and power motive). Because achievement motive scores were significantly correlated with the number of words in the stories (achievement motive: *r* = 0.41, *p* < 0.05; power: *r* = 0.03, *p* = 0.86; affiliation: *r* = 0.28, *p* = 0.14), they were residualized for word count (for this procedure, see Schultheiss and Pang, 2007). All motive scores were z-standardized for further analyses. Detailed information about test administration, motive coding, correction for protocol length and about the PSE’s reliability and validity is given in Schultheiss and Pang (2007). Administration of the PSE as an online version is described in Bernecker and Job (2011).

Trait self-control was assessed using a German version of the Self-Regulation Scale (SRS, Schwarzer et al., 1999). It consists of 10 items that capture dispositional control in goal pursuit (e.g., I stay focused on my goal and do not allow anything to distract me from my plan of action. If an activity arouses my feelings too much, I can calm myself down so that I can continue with the activity soon). Participants are asked to indicate their (dis)agreement to each statement using a rating scale ranging from 1 (I fully agree) to 4 (I fully disagree). We calculated the mean of all 10 items to operationalize trait self-control. In previous research, the SRS showed satisfactory test-retest reliability, good internal consistency, and showed cross-cultural criterion-validity by predicting measures such as self-efficacy, proactive coping, and positive affect (Luszczynska et al., 2004; Diehl et al., 2006).

Motor performance was operationalized by assessing participants’ hand-grip strength. Participants were asked to press a dynamometer (Vernier, Beaverton, OR, USA) as hard as possible for the duration of a 5-s beep signal with their dominant hand (10 times for each of four blocks). The measurement of handgrip strength was recorded in Newton and saved *via* the software LoggerLite, version 1.9.2 (Vernier, Beaverton, OR, USA).

DLPFC-activation was measured using functional near-infrared spectroscopy (fNIRS; System NIRSport, NIRx Medical Technologies, LLC, New York, NY, USA). fNIRS quantifies the degree of dLPfC oxygenation changes by detecting local changes in hemoglobin concentration. Local differences in hemoglobin concentration are used as proxy for changes in cortical activity since neuronal activity requires energy, which is provided by subsequent delivery of oxygenated blood. Hemoglobin is the relevant oxygen transport molecule, named oxyhemoglobin (O_2_Hb) when oxygenated and deoxyhemoglobin (HHb) when deoxygenated. For the fluctuation measurement of O_2_Hb and HHb, fNIRS makes use of the fact, that human tissue is relatively permeable for light in the near-infrared spectrum (700 nm to 1,400 nm, NIR; Jöbsis, 1977; Ferrari and Quaresima, 2012). Therefore, this specific span of wavelengths is called “optical window” (Jöbsis, 1977). In this frequency spectrum, O_2_Hb and HHb exhibit clearly differentiating spectra of absorption. For measurement of the cerebral cortex blood flow, NIR light is emitted in two wavelengths (e.g., 760 nm and 850 nm) into the human tissue. Detectors are measuring how much light of each wavelength is exiting the tissue. Relative differences in received light serve as proxy for changes in blood flow and differences in the ratio of O_2_Hb and HHb in the examined area (Ferrari and Quaresima, 2012).

**Figure 2:**
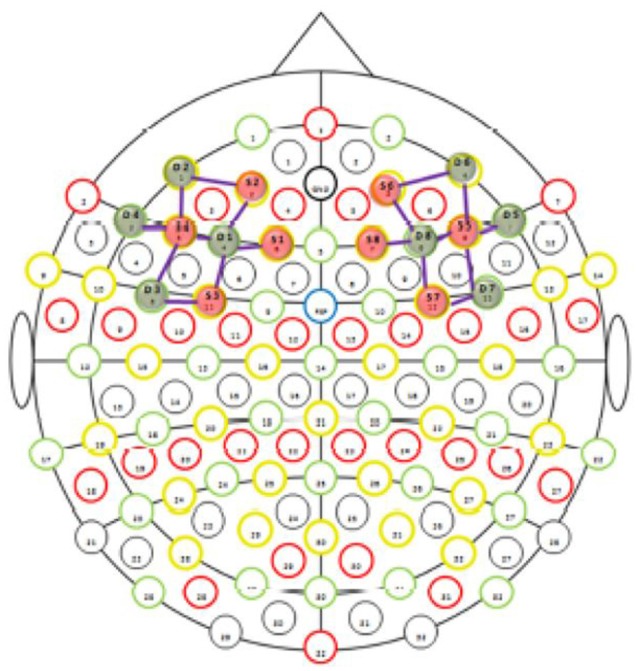
Dorsolateral prefrontal cortex (dLPFC) montage (NIRxMedical Technologies LLC, 2016). For the functional near-infrared spectroscopy (fNIRS) measurement, emitters (E) and detectors (D) were positioned according to the international 5/10 system: E1 at F1, E2 at AF3, E3 at FC3, E4 at F5, D1 at F3, D2 at AF7, D3 at FC5, D4 at F7, E5 at F6, E6 at AF4, E7 at FC4, E8 at F2, D5 at F8, D6 at AF8, D7 at FC6, and D8 at F4.

In our study, we assessed oxygenation of the prefrontal cortical region which is commonly referred to as dLPFC (Brodmann’s area 8, 9, 46). Figure 2 shows the exact optode placement in our study.

## Results

### Missing Data

From the 28 participants, one participant had to discontinue the experiment after data-collection of two experimental blocks, due to an acute migraine onset. For one participant no handgrip data was recorded due to a false presetting of the LoggerLite software. Nine complete fNIRS recordings could not be analyzed due to different technical reasons^2^. Missing values were not imputed but excluded from statistical analysis. Thus, final sample sizes differ for goal conditions and for the two outcome variables handgrip strength, and dLPFC oxygenation (fNIRS measurements right and left dLPFC: calibration goal: *n* = 19, achievement goal: *n* = 18; power goal: *n* = 18; affiliation goal: *n* = 20; handgrip strength data: calibration goal: *n* = 27; achievement goal: *n* = 26; power goal: *n* = 26; affiliation goal: *n* = 27).

### Pre-processing of Data

fNIRS data were preprocessed using HOMER2 vs. 2.2 (Huppert et al., 2009; MathWorks Inc., 2016). For each subject, the *enPruneChannels* function was used with the following function arguments to remove channels when the signal was too weak or too strong: *dRange*_(1)_ = 1e^−2^; *dRange*_(2)_ = 3e; *SNRthresh* = 2; *SDrange*_(1)_ = 0.0; *SDrange*_(2)_ = 45.0, *reset* = 0. Then, optical intensity was converted to optical density using the *Intensity to OD* function. To remove motion artifacts, the *Wavelet_Motion_Correction* was run with an IQR of 1.5 (Molavi and Dumont, 2012). This method entails a discrete wavelet transformation and is efficient in recovering the hemodynamic response function (HRF; Brigadoi et al., 2014). Subsequently, motion artifacts were removed using the *hmrMotionArtifact* function and function arguments were specified as follows to mask any drastic signal changes: *tMotion* = 0.5; *tMask* = 1.0; *STDEVthresh* = 10.0; *AMPthresh* = 1.00. Triggers representing cue-words within trials were rejected if they fell within a time range of −5 to +10 s of a so-detected motion artifact (*enStimRejection: tRange*_(1)_ = −5.0; *tRange*_(2)_ = 10.00). Then, data were low pass filtered (0.5 Hz) and converted to oxyhemoglobin (O_2_Hb) with the modified Beer-Lambert law (Delpy et al., 1988). For this conversion, standard path length factors were used and set to 6.0 (for 760 nm) and 6.0 (for 850 nm). For each participant, four different trigger types had been set during fNIRS recording (NIRStar, RRID: SCR_014540) to each represent cue words of one single experimental block/goal condition. A last step of fNIRS preprocessing used the *hmrBlockAvg* function to average oxyhemoglobin values over all trials belonging to an experimental block. *HmrBlockAvg* maintained oxyhemoglobin values within a time segment of 2 s before and 10 s after trigger-set.

Preprocessed data of individual participants was exported from HOMER2 into R-Studio (RStudio Team, 2017). In R-Studio only oxyhemoglobin values within a time segment of 5–7 s after trigger-set were averaged, because stimulus-induced hemodynamic responses were shown to peak within this interval (Huppert et al., 2006). Those means were again averaged for all channels representing dLPFC oxygenation.

### Statistical Approach for Testing Hypotheses

In order to test the two-way interaction hypotheses according to which participants with a strong achievement motive show lower dLPFC oxygenation and better performance, respectively, in the achievement goal condition than in the motive-incongruent conditions, we conducted three types of comparisons. We contrasted the achievement goal condition against the motive-neutral calibration condition, the achievement goal condition against the power condition and the achievement goal condition against the affiliation condition, respectively. So three separate hierarchical multi-level regression analyses (RStudio, Bates et al., 2015) were conducted for each of the dependent variables (dLPFC oxygenation, motor performance).

The predictor variables, the z-standardized achievement motive score (residualized for word count) and the goal conditions (dummy-coded 1: achievement account vs. 0: calibration account/or power account/or affiliation account) were the between-subject variables in this model. The dLPFC oxygenation is a continuous, and interval-scaled dependent variable. We used a nested random effect regression as a multi-level regression approach that allows the intercept to vary with participants. The goal condition variable was nested within participants. The model was built by sequentially adding predictors after an intercept-only model and its random intercept (random = 1 | ID) had been specified. Every newly added predictor or interaction-term was stored as a separate regression model in R-studio to allow for a final comparison of all stages of model-specification. All predictors were kept in a regression model when a further predictor or interaction-term was added to that model. To test the Motive × Goal interaction hypotheses on dLPFC oxygenation (DVs were not standardized), the single predictors (step 1: AchMotive, step 2: Goal Condition) were consecutively added to the regression model. In a third step, the two-way interaction term was added to the model.

In order to examine the effects on handgrip, first handgrip strength was baseline-corrected (baseline handgrip strength was subtracted from handgrip strength in experimental trials) and then used as a dependent variable in hierarchical multi-level regression analysis. We had 10 data points per Block (10 trials, see Figure 1) and computed a random intercept/slope regression with the intercept specified to vary with participants and the slope for Goal Conditions within participants (1: achievement, 0: calibration account/or power/or affiliation account, respectively). The model was similarly built up in a hierarchical, forward-stepwise fashion by sequentially adding predictors after an intercept-only model and its random parts (random = Goal_Condition | ID) had been specified.

In order to test the three-way interaction hypothesis (Motive × Goal condition × Trait Self-control), according to which participants with a strong achievement motive who additionally have strong trait self-control show less impairment of motor performance in the motive-incongruent than in the congruent conditions, we extended the statistical model and additionally included trait self-control (z-standardized), the two-way interactions (Motive × Self-control and Self-control × Goal condition) and the three-way interaction (Motive × Goal condition × Trait Self-control) into the hierarchical multilevel model. We built the same model when examining the influence of trait self-control on dLPFC-oxygenation in motive-congruent and motive-incongruent conditions.

### Descriptive Statistics

Table 1 displays means and standard deviations for the achievement motive, trait self-control and for handgrip strength. Scores for dLPFC oxygenation and hand-grip performance during the experiment are mean scores across all conditions. In accordance with previous findings (Gonzales and Scheuermann, 2007), hand grip performance decreases from the baseline measure to the end of the experiment due to fatigue effects. Pearson correlations revealed that the achievement motive raw score was significantly positively correlated with trait self-control (*r* = 0.37, *n* = 28, *p* < 0.05) and negatively correlated with dLPFC oxygenation (*r* = −0.49, *n* = 28, *p* < 0.05). Correlation coefficients with the word-count corrected motive score were slightly smaller (see Table 1).

**Table 1:** Means, standard deviations and correlations (Pearson, two-tailed) for achievement motive, dispositional self-control, dorsolateral prefrontal cortex (dLPFC)-activation and hand grip performance. The latter two variables are averaged across all conditions.

	1	2	3	4	5	M	SD
1 ACH Motive	-	0.91***	0.37*	−0.49*	−0.18	4.11	2.42
2 ACH Motive_res^1^	-	-	0.39*	−0.38	−0.22	0.00	2.22
3 Disp. self-control	-	-	-	−0.15	−0.25	2.77	0.25
4 dLPFC activation^2^	-	-	-	-	0.23	0.004	0.078
5 Hand-grip performance^3^	-	-	-	-	-	214.67 (−34.70)	58.56 (36.92)

Note.*p < 0.05. ***p < 0.001. ^1^Residualized motive score (word count corrected). ^2^Scores for dLPFC activation represent cortical oxygenation (mean of 5–7 s after trigger). ^3^Hand-grip performance scores are displayed in Newton. Statistical analysis included baseline-subtracted hand-grip performance scores for individual participants. Mean and standard deviation for baseline-subtracted values are indicated in brackets.

### Test of Hypotheses

Table 2 displays details for the two-way interaction analyses. Contrary to our hypotheses, the hierarchical multi-level regression analyses revealed no significant Motive × Goal condition interaction effects in the prediction of dLPFC oxygenation. Similarly, none of the three analyses testing the effects of motive-goal incongruence on hand-grip performance revealed significant Motive × Goal interactions.

**Table 2:** Results of the two-way interaction analyses (Motive × Goal condition) for dLPFC oxygenation (upper part) and motor performance (lower part of table).

Types of goals	ACHMotive	Goal	ACHMotive × Goal
dLPFC oxygenation
ACH vs. CAL	value: −0.020, *SE* = 0.030 *t*_(17)_ = −0.651, *p* = 0.052	value: −0.055, *SE* = 0.045 *t*_(16)_ = −01.211, *p* = 0.24	value: −0007, *SE* = 0.043 *t*_(16)_ = 0.170, *p* = 0.867
ACH vs. POW	value: −0.065, *SE* = 0.023 *t*_(16)_ = −2.862, *p* = 0.011	value: −0.005, *SE* = 0.033 *t*_(16)_ = −0.146, *p* = 0.890	value: 0.053, *SE* = 0.032 *t*_(16)_ = 1.643, *p* = 0.120
ACH vs. AFF	value: −0.010, *SE* = 0.024 *t*_(18)_ = −0.417, *p* = 0.682	value: −0.014, *SE* = 0.036 *t*_(16)_ = 0.386, *p* = 0.705	value: −0.003, *SE* = 0.034 *t*_(16)_ = −0077, *p* = 0.939
Motor performance
ACH vs. CAL	value: 9.128, *SE* = 8.045 *t*_(25)_ = 1.134, *p* = 0.267	value: 3.711, *SE* = 5.873 *t*_(501)_ = 0.631, *p* = 0.528	value: 8.273, *SE* = 5.931 *t*_(501)_ = 1.395, *p* = 0.164
ACH vs. POW	value: 12.543, *SE* = 7.155 *t*_(24)_ = 1.753, *p* = 0.092	value: −2.839, *SE* = 4.378 *t*_(492)_ = −0.649, *p* = 0.517	value: 5.527, *SE* = 4.431 *t*_(492)_ = 1.247, *p* = 0.213
ACH vs. AFF	value: 10.993, *SE* = 6.862 *t*_(25)_ = 1.602, *p* = 0.122	value: −0.901, *SE* = 4.263 *t*_(501)_ = −0.211, *p* = 0.833	value: 5.680, *SE* = 4.313 *t*_(501)_ = 1.317, *p* = 0.189

*Notes. ACH, achievement goal condition; CAL, calibration condition; POW, power goal condition; AFF, affiliation goal condition*.

**Figure 3:**
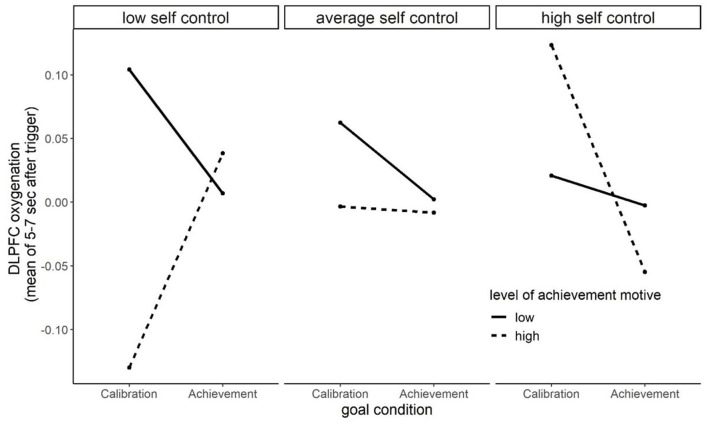
Illustration of the marginal ACHmotive × Goal (achievement vs. calibration goal) × Trait Self-control interaction effect on DLPFC oxygenation (μmol/L).

Table 3 displays the results of the three-way interaction analyses. It shows one marginal Motive × Trait Self-control × Goal condition interaction predicting dLPFC oxygenation when testing the achievement goal condition against the motive-neutral calibration. The pattern of the marginal interaction is displayed in Figure 3. It shows that for people with weak achievement motives (solid lines) the dLPFC oxygenation depended less on the strength of trait self- control (regardless of motive-goal congruence). In contrast, dLPFC responses of participants with strong achievement motives (dotted lines) are highly sensitive to levels of trait self-control. For high self-controllers (right part of Figure 3) the interaction pattern was as assumed in our two-way-interaction hypothesis: Participants with strong achievement motives showed higher dLPFC oxygenation in motive-incongruent than in motive-congruent goals. What warrants discussion (see below), however, is that the interaction pattern for low trait self-controllers (left part of Figure 3) shows exactly the opposite. Here, participants with a strong achievement motive had lower dLPFC oxygenation in the incongruent calibration than in the congruent achievement goal group.

**Table 3:** Three-way interactions (Motive × Trait Self-control × Goal condition) to predict dLPFC oxygenation (upper part) and motor performance (lower part of table).

Types of goals	Motive × Trait Self-control × Goal condition		
	value	SE	*t*(df), *p*
**dLPFC oxygenation**
ACH vs. CAL	−0.106	0.057	*t*_(14)_ = −1.875, *p* = 0.082
ACH vs. POW	−0.056	0.043	*t*_(14)_ = −1.290, *p* = 0.218
ACH vs. AFF	−0.002	0.045	*t*_(14)_ = −0.047, *p* = 0.963
**Motor performance**
ACH vs. CAL	−13.487	6.683	*t*_(499)_ = −2.018, *p* = 0.044
ACH vs. POW	1.003	5.232	*t*_(419)_ = −0.192, *p* = 0.848
ACH vs. AFF	2.025	5.073	*t*_(499)_ = 0.400, *p* = 0.690

*Notes. ACH, achievement goal condition; CAL, calibration condition; POW, power goal condition; AFF, affiliation goal condition*.

**Figure 4:**
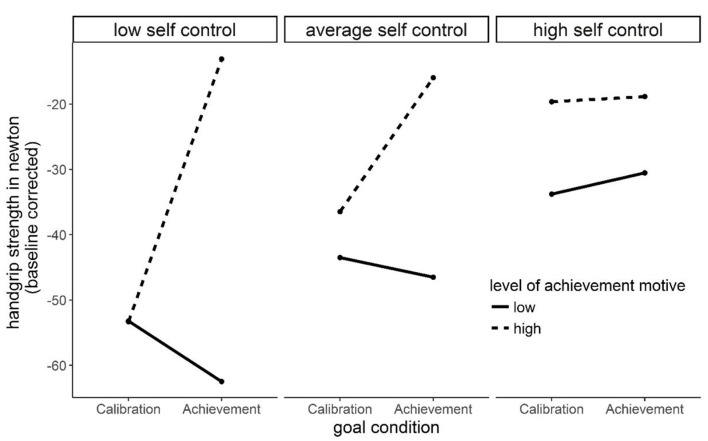
Illustration of the significant ACHmotive × Goal (achievement vs. calibration goal) × Trait Self-control interaction effect on handgrip performance (in Newton, baseline corrected).

A significant three-way interaction was found when predicting handgrip performance. Figure 4 displays the interaction pattern. Reading it from left to right (low to high trait self-control), the slope becomes considerably flatter for participants with a strong achievement motive due to an increase of performance in the motive-incongruent calibration group. To a much weaker extent, this pattern of result is also true for individuals with a weak achievement motive. This interaction pattern is in accordance with our assumption that high trait self-control can compensate for performance impairment caused by motive-incongruent goals.

## Discussion

One important gain of insight of the present study is based on non-significant results: none of our two-way-interaction hypotheses were supported. The achievement motive did not interact with congruent (achievement) and incongruent (power, affiliation, and calibration) goals when predicting motor performance and dLPFC oxygenation. One reason might be that motor performance (e.g., other manual tasks such as fingers coordination) and cortical correlates of self-control can be operationalized in a more suitable fashion. Another interpretation of the non-significant two-way interactions is that the effects of motive-goal incongruence on motor performance and dLPFC oxygenation only then become visible when trait self-control is additionally considered. Unlike well-being and ill-being that are directly predictable from motive-goal (in)congruence (Brunstein et al., 1995, 1998; Hofer and Chasiotis, 2003; Baumann et al., 2005; Brunstein, 2010b; Hofer et al., 2010; Job et al., 2010; Langan-Fox and Canty, 2010; Pueschel et al., 2011; Hofer and Busch, 2017; see above, e.g., Schüler et al., 2009), motor performance and cortical correlates of state self-control require a more complex explanatory model. Trait self-control influences how motive-goal incongruence exerts its influence on hand grip strength and dLPFC oxygenation. Directly referred to our data, the significant three-way interaction effect on motor performance showed that achievement motive-goal incongruence leads to impairment of motor performance in people with low trait self-control. High trait self-control, however, can compensate for the impairment of motor performance caused by motive-incongruence (see Figure 4).

It was, however, unexpected that the three-way interaction to predict dLPFC oxygenation did not reach conventional levels of significance and revealed an interaction pattern that requires some discussion. When trait self-control is high (right part of Figure 3), individuals with a strong achievement motive showed lower dLPFC oxygenation in the congruent achievement goal condition than in the incongruent calibration condition. This is a pattern that we would have expected in our two-way interaction hypotheses. However, the interaction pattern was precisely the opposite for people with low trait self-control (left part of Figure 3). So, we either have to question trait self-control as a compensatory tool that can outweigh negative consequences of motive incongruence or question our dependent variable. The “self-control as compensation” approach was nicely supported when predicting motor performance and is in accordance with the more general finding that self-control is associated with performance (e.g., Duckworth and Seligman, 2005; de Ridder et al., 2012; Englert, 2017). Therefore, we feel that we have to critically rethink our dependent variable “dLPFC oxygenation” and the role it plays in self-control and allow us the following speculations. As briefly introduced in the hypotheses section above, we originally viewed dLPFC oxygenation as an indicator of self-control demands that are imposed by a congruent or incongruent task. However, viewing the role of the dLPFC in *resolving* rather than in *detecting* conflicts (the latter is a task that the dACC is presumably responsible for; e.g., Botvinick et al., 2001, 2004; Shenhav et al., 2017), a different interpretation becomes plausible. DLPFC oxygenation would then represent the resolving of a conflict, for example by investing effort when being confronted with a challenging task (hand grip) rather than giving up. In other words, in our study, we might have assessed the actual exerted self-control in a strenous activity rather than the self-control requirements of a congruent or incongruent task. For people with a strong achievement motive, who per definition prefer challenging tasks, self-control is an indispensable means-to-an-end (achieving high performance standards). With regard to our study and the interaction pattern, one could assume that individuals with a strong achievement motive (and low dispositional self-control) are willing to exert self-control when their effort in a challenging and strenous task (hand grip task) serves a motive-congruent goal (e.g., achieving a high individual performance standard) and less willing to invest effort, when the mean (effortful self-control) does not fit the “end.” In other words, a calibration goal is not worth to put effort in because it does not promise the satisfaction of the achievement motive. People with high trait self-control, in contrast, can override this motive guided investment of effort, for example in situations in which effort is expected from the social environment (e.g., from the experimenter, teacher, sport-coach). In brief, trait self-control enables people to act against their implicit motives or to compensate for a lack of motive-relevant incentives. Referring to our three-way-interactions, trait self-control enables to initiate mechanisms (e.g., make state self-control available) and behavior (putting effort in strenuos tasks) that are not supported by an anticipated affective reward (motive satisfaction), but are necessary to attain a goal (fulfill the requirements of the experimenter).

Viewing both interaction patterns in a temporal sequence also speaks in favor of this interpretation. Individuals with strong achievement motives and low dispositional self-control perform worse in a calibration task because they exert less state self-control (left parts of Figures 3, 4). In contrast, high dispositional self-control makes state self-control available that in turn leads to better performance (right parts of Figures 3, 4). In sum, trait self-control compensates for a lack of motive-relevant incentives by making state self-control available. Undoubtedly, these theoretical considerations are highly speculative *post hoc* interpretations of unexpected results and have to be treated with the utmost caution. However, they are in accordance with conclusions drawn by other researchers that implicit motives moderate the exertion of self-control in motive-related tasks (Gröpel and Kehr, 2014) and in accordance with the understanding of self-control as being depending on motivational factors (Kehr, 2004b; Inzlicht and Schmeichel, 2012).

Also unexpected (in the sense of not being formulated in a hypothesis) correlational analyses across all goal conditions showed that the achievement motive was significantly negatively correlated with dLPFC—activation. This is fully in conformance with the theoretical framework of the achievement motive (McClelland et al., 1953; Brunstein and Heckhausen, 2008) and the reasoning behind the present research that people with a strong achievement motive need less self-control in (experimental) settings that fit their motive. This certainly applies to the achievement-related task (performing well in a challenging task) in our experiment.

That we found interaction effects when contrasting the effects of an achievement goal against the effects of a motive-neutral goal (calibration condition) is encouraging. That we did not find significant interaction effects when contrasting achievement goals against power and affiliation goals, respectively, warrants discussion. A more detailed look into Winter’s research provides an explanatory approach. Winter (1996) distinguishes into two types of motive incongruence. First, a person sets a goal that is not backed up by a corresponding motive. According to (Brunstein, 2010a, p. 244), this requires “to boost the incentive value of a goal that is not very attractive in its own right […].” In our study, this is the case when people with a strong achievement motive have to strive for a motive-neutral calibration goal that frames the task as a routine task rather than as an achievement task. In this case, *impulse control* means “*generating”* an impulse for action. The second type of motive incongruence occurs when striving for a goal is in direct conflict with a motive in another domain. Here it is necessary to control impulses, in the sense of *“suppressing”* impulses, that emanate from the motive, because it otherwise would impede the realization of the goal (Brunstein, 2010a, p. 245). In our study, this is the case when participants with a strong achievement motive have to suppress impulses and strive for an affiliation or power goal. These considerations about motive incongruence have obvious parallels with genuine self-control research that also distinguish into two facets of self-control (*action initiation* vs. *action inhibition*; see de Ridder et al., 2012). Self-control comprises the *initiation* of an unpleasant action (e.g., follow a disliked exercise regime/strive for a goal that is not affectively-charged by a corresponding motive) as well as to *suppress* an unwanted action impulse (e.g., not to eat chocolate when wanting to keep to a diet/striving for a goal that requires to act against an implicit motive).

Referring to our study, we speculate that with our study design we did not appropriately address the second facet of motive-incongruence that is activity inhibition. Doing one’s best for a team (affiliation goal instruction) or showing one’s performance superiority (power goal instruction) in our hand grip task does not necessarily contradict the impulse of performing better that is derived by the implicit achievement motive. Therefore, the other goal conditions might not have directly required to suppress the impulse to perform better. In order to better address impulse-control (in the sense of suppressing an impulse), a true and pure conflict between the achievement motive and a goal has to be experimentally created in creative experimental (but realistic) settings. The achievement motive could, for example, be incited by a challenging task of moderate difficulty including immediate feedback (e.g., online tracking of one’s handgrip strength on the computer screen), but participants then are asked to suppress their impulse to outperform themselves but instead hold back performance in order to attain an affiliation-related goal (e.g., cover story: underperformance is necessary to maintain a harmonious and friendly atmosphere in a working team). Furthermore, because all goal conditions allow the collection of hand grip points, task indicators of performance were present in all conditions and therefore might have triggered achievement behavior of participants with a strong achievement motive (even though to a lesser extent).

Furthermore, our operationalization of self-control can be broadened in future studies. DLPFC oxygenation is just one out of many possible cortical correlates of one out of different facets of self-control. As already indicated above, the dACC is responsible for detecting conflicts (Kerns et al., 2004) and therefore its activation might be an even better operationalization for conflicts raised by motive incongruence. Assessing the oxygenation of both, dLPFC and dACC could help to disentangle the detection of a conflict (e.g., stronger oxygenation of dACC in motive incongruent goal conditions) and the attempt to resolve this conflict (dLPFC).

Our research approach also raises questions for the applied domain. We used an artificial laboratory context to experimentally-induce motive-congruent goals. However, striving for goals that are assigned by others is also common practice in natural settings, for example at the workplace when the boss declares the future business objectives, or in sports when the coach gives instructions and sets goals for the next season and even in the family when parents have more or less explicitly stated expectations and career goals for their children. When these goals do not match with the employee’s, the athlete’s or the child’s implicit motives, self-control might be required. Striving for long-term goals (e.g., sport or academic career) even require a permanent use of self-control. Chronic self-controlled actions are accompanied with unpleasant feelings (e.g., exertion) and therewith can impair subjective well-being (e.g., at the workplace, Kehr, 2004a,b) and athletic performance (Englert, 2017) in the long run (see also strength model of self control, Baumeister et al., 1994, 1998). Our experimental study needs support from field studies with a higher ecological validity that is better able to capture the complexity of human life.

To conclude, although trait self-control is indispensable for adaptive behavior and associated with a wide variety of positive life outcomes (Tangney et al., 2004; de Ridder et al., 2012), it might be even better to prevent self-control dilemmas right from the beginning by setting motive-congruent goals. This should avoid the negative consequences of motive-incongruence (Kuhl, 2001) and paves the way for intrinsic motivation. So, whenever having the choice to self-set one’s goals, motive-congruence should be a declared objective. Encouraging strategies to enhance the congruence of self-set goals to one’s implicit motives have already been developed, for example using goal imagery (Schultheiss and Brunstein, 1999) and focussing on affects when fantasizing about one’s goals (Job and Brandstätter, 2009). Our results, however, suggest that two ingredients make up the perfect mixture: Motive congruent goal setting, which leads to well-being, makes self-control capacity easier available and results in high performance as well as high trait self-control that helps to compensate motivational deficits in phases of goal striving in which motive-relevant incentives are less strong. Previous research has already provided support for both ways to success that we recommend to combine: congruence-enhancement training (Roch et al., 2017; see also Schultheiss and Brunstein, 1999; Job and Brandstätter, 2009) and self-control trainings to built up one’s self-control capacity (Muraven, 2010; Job et al., 2016).

At the end of the discussion, we would like to note again that our study is a first attempt to consider cortical correlates of self-control due to motive-goal incongruence and therefore certainly has its weaknesses. One limitation is the small sample size that is unproblematic for direct effects (due to the within-subject design of the study), but clearly disputable for the interaction hypotheses. Therefore, we ask the readers to treat the results with care. We hope, however, that with publishing this study as a pilot study we can open a broader discussion about the necessity to consider individual differences and their interactions with situational characteristics (e.g., goal instructions) when assessing cortical correlates of self-control in strenuous tasks. We furthermore would like to invite motive researchers to adopt more experimental approaches to motive-goal incongruence research. Here, we have extended motive-goal incongruence research in regard to the chosen methodological approach (experimentally-induced goals in the laboratory rather than goal assessement in everyday life) and the dependent variables that were assessed (assessement of performance rather than subjective ratings of well-being; cortical correlates of self-control). We would like that our interpretation of the results invites fellow researchers to join us in this line of research.

## Data Availability Statement

The raw data supporting the conclusions of this manuscript will be made available by the authors, without undue reservation, to any qualified researcher.

## Ethics Statement

Ethical review and approval was not required for the study on human participants in accordance with the local legislation and institutional requirements. The patients/participants provided their written informed consent to participate in this study.

## Author Contributions

JS, JH, and WW contributed to the conception, and to the planning of the study (study design, material). JH and WW collected data and organized the database. JH and JS performed the statistical analysis. JS wrote the first draft of the manuscript. JH and WW wrote sections of the manuscript. All authors contributed to manuscript revision, read and approved the submitted version.

## Conflict of Interest

The authors declare that the research was conducted in the absence of any commercial or financial relationships that could be construed as a potential conflict of interest.

## Footnotes

^1^Further variables that were not part of the present research question were measured. We used the study to additionally capture endocrine responses to motive-goal incongruence and assessed salivary alpha amylase. This variable, as well as the stimuli valence (assessed using the Self-Assessment Manikin) were part of a different, however, related research question.

^2^For two complete fNIRS recordings, the overall signal quality was too noisy to be useful for data analysis. Even artifact correction via the utilized processing stream could not recover valid data from those two fNIRS recordings. For four initial fNIRS recordings, the synchronization with the experimental manipulation was tried to be established by starting fNIRS and the experimental PsychoPy script at the same time. This method of synchronization proved to be unsuccessful, as it turned out that NIRstar was already starting the recording of the time series, when the signal calibration was previewed. Postprocessing of those four fNIRS time series did not allow the isolation of those segments that occurred in synchrony with experimental manipulations. Three other complete fNIRS recordings were lost due to a software error of NIRstar on one single day of data-collection. For yet unsettled reasons, NIRstar did not convert the fNIRS recordings into nirs-files, which constitutes the required file-format for all further steps of data-processing in HOMER vs. 2.2. Any attempts to convert available NIRstar output-files from those three fNIRS recordings into the required nirsfile format, were not successful.
